# New Adhesive Dressing

**Published:** 1850-09

**Authors:** 


*New Adhesive Dressing.* By Dr. Mellez.—From his dissatisfaction with the ordinary adhesive dressings, Dr. Mellez was led to adopt a solution of "gum lac," which is much used in the arts as a varnish and adhesive.

He uses a solution of this substance in spirit of wine, made with the aid of a moderate heat, and in such proportion as to give a mixture having the consistence of jelly, or nearly approaching to it. It can be made in a common wide-mouthed bottle, and a simple cork suffices for its preservation. When he uses it, he spreads it with a spatula upon pieces of cloth or taffetas, cut to the size required. According to him, it possesses all the good qualities of collodion, and even in a higher degree than that substance—viz., contraction during its dessication, impermeability to air, absence of all irritating action on the skin or wound, intimate adherence to the skin, and resistance to the action of water, fatty matters, or the discharges from the wound. It has not, like collodion, the quality of being colourless; but he believes that it might, if required, be decolorized, and, if applied upon animal membrane, would furnish a transparent dressing. It does not dry so quickly as collodion, but that is the only advantage the latter possesses. The lac dressing, however, does not require so long time as to be inconvenient to the surgeon. Moreover, it is not indispensible for the lac, as it is for the collodion, that the skin be absolutely dry. It has further, the advantage (not to be overlooked) of being more moderate in cost. ('Bulletin de Théraputique.')—*Monthly Journal, June* 1850, *p.* 575.

